# The College of Nursing's Traducers

**Published:** 1919-11-22

**Authors:** 


### THE COLLEGE OF NURSING'S TRADUCERS.

There is a type of mind and a habit of writing which may sow injustice by inaccurate or unfounded statements continued to any extent. We have had in the past week two examples of this habit of inaccurate utterance. One of the inaccurates is Miss Maud MacCallum, a member of the Nurses' Co-operation, who describes herself as honorary secretary of the newly-formed Trades Union of Nurses. Miss MacCallum declares that "the College of Nursing does nothing to advance the material interests of the working nurse. It is really an employers' association." We are fortunate in being able to publish most striking proof that Miss MacCallum's statement is fiction concerning the College, in an important letter issued on the 17th inst. by the Hon. Sir Arthur Stanley, G.B.E., the Chairman of the College, see page 168. He gives a remarkable list of the good things the College has obtained for trained nurses since its establishment four years ago. So far from doing nothing, it has accomplished much, as every one of our readers may see and judge for themselves by referring to this letter.

Miss MacCallum is not content with her honorary office as a promoter of a Trades Union for nurses, but seems out seriously to damage, and, as we understand by her actions, to destroy if possible, the greatest Nurses' Co-operation in the world. She has been a member of that organisation, we understand, for twenty years, and during that time she must have reaped great benefit from the excellent management of its affairs by the general committee, of which she is a member. So opposed to the interests of the members of the Nurses' Co-operation is Miss MacCallum's action that we have no doubt the members will combine and save their property from the wreckage with which it is at present threatened.

Miss MacCallum properly condemns hospitals which are increasing their private staff and competing unfairly with the working nurse. She gives no instances, and whatever they may be they must be few in number, but the abuses where they exist, however insignificant the employers may be, should be immediately exposed by publication in the Press. Miss MacCallum states that there are nursing homes which charge very big fees for sending nurses out, but pay very small ones to the nurses sent. We hear of others who have sent out as nurses in uniform women who have no claim or right to pose as a nurse. The County Councils or Ministry of Health, or both, should regularly inspect all Nurses' Homes and Supply Agencies.

Why do not the nurses combine, and send us for publication the names and addresses of every so-called nursing home that pursues the abominable practice of sweating nurses in the way described? The abuse should then be promptly ended. As to the establishment of a nurses' trades union, it is impossible for anyone who is familiar with the rules and practices of these organisations and has a knowledge of trained nurses of the best type, to believe, that any number of trained nurses can have so small an intelligence and regard for their own interests, as to suffer themselves to fall victims to the injury and ultimate loss of employment, which membership of a trades union may bring about.

